# Reduction in Acquisition Time and Improvement in Image Quality in T2-Weighted MR Imaging of Musculoskeletal Tumors of the Extremities Using a Novel Deep Learning-Based Reconstruction Technique in a Turbo Spin Echo (TSE) Sequence

**DOI:** 10.3390/tomography8040148

**Published:** 2022-07-06

**Authors:** Daniel Wessling, Judith Herrmann, Saif Afat, Dominik Nickel, Ahmed E. Othman, Haidara Almansour, Sebastian Gassenmaier

**Affiliations:** 1 Department of Diagnostic and Interventional Radiology, University Hospital of Tuebingen, 72076 Tuebingen, Germany; daniel.wessling@med.uni-tuebingen.de (D.W.); judith.herrmann@med.uni-tuebingen.de (J.H.); haidara.al-mansour@med.uni-tuebingen.de (H.A.); sebastian.gassenmaier@med.uni-tuebingen.de (S.G.); 2 MR Application Predevelopment, Siemens Healthcare GmbH, 91052 Erlangen, Germany; marcel.nickel@siemens-healthineers.com; 3 Department of Diagnostic and Interventional Neuroradiology, University Hospital of Mainz, 55131 Mainz, Germany; ahmed.e.othman@googlemail.com

**Keywords:** deep learning, accelerated turbo spin echo MRI, musculoskeletal imaging, musculoskeletal tumors, artificial intelligence

## Abstract

Background: The aim of this study was to assess the technical feasibility and the impact on image quality and acquisition time of a deep learning-accelerated fat-saturated T2-weighted turbo spin echo sequence in musculoskeletal imaging of the extremities. Methods: Twenty-three patients who underwent MRI of the extremities were prospectively included. Standard T2w turbo inversion recovery magnitude (TIRMStd) imaging was compared to a deep learning-accelerated T2w TSE (TSEDL) sequence. Image analysis of 23 patients with a mean age of 60 years (range 30–86) was performed regarding image quality, noise, sharpness, contrast, artifacts, lesion detectability and diagnostic confidence. Pathological findings were documented measuring the maximum diameter. Results: The analysis showed a significant improvement for the T2 TSEDL with regard to image quality, noise, contrast, sharpness, lesion detectability, and diagnostic confidence, as compared to T2 TIRMStd (each *p* < 0.001). There were no differences in the number of detected lesions. The time of acquisition (TA) could be reduced by 52–59%. Interrater agreement was almost perfect (κ = 0.886). Conclusion: Accelerated T2 TSEDL was technically feasible and superior to conventionally applied T2 TIRMStd. Concurrently, TA could be reduced by 52–59%. Therefore, deep learning-accelerated MR imaging is a promising and applicable method in musculoskeletal imaging.

## 1. Introduction

Tumors of the extremities comprise a wide range of pathologies. Musculoskeletal tumors, including rare clear cell sarcoma, alveolar sarcoma, and epithelioid sarcoma, and more common tumor entities such as Ewing sarcoma and osteosarcoma account for approximately 8% of all malignancies in young adults [1]. There is a wide variety of histological subtypes in soft tissue tumors [2], which makes a purely image-based diagnosis almost impossible in many cases. Magnetic resonance imaging (MRI) and computed tomography (CT) are the state-of-the-art imaging modalities to be used to evaluate tumor composition and T-staging regarding the possible involvement of adjacent anatomical structures [3]. As MRI provides a better soft tissue contrast, and therefore, allows a more thorough differentiation of the intrinsic tumor characteristics and the local extent, it is the imaging of choice in musculoskeletal tumors.

In recent years, new methods have been developed to reduce MRI acquisition time as well as the extent of artifacts and to achieve more precise imaging [4,5,6]. Recently, machine learning and artificial intelligence-based algorithms have found their way into clinical radiological imaging [7,8]. These deep learning algorithms (DL) are based on convolutional neural networks (CNN) that were developed on the basis of the function of animal neurobiology, resembling the human neural network [9]. Deep learning is focused on automatized feature learning [10]. The process is based on automated learning processes and stated hyperparameters [11]. CNN kernels are used in convolutional networks to extract important image features and create algorithms throughout the image. In a pooling process, unnecessary data are discarded without any negative impact on the final result [11,12].

In radiology, trained algorithms based on larger datasets have primarily been introduced in, e.g., classification, segmentation, pattern recognition, and artificial intelligence-based diagnosis [13,14]. In the meantime, the inclusion of these components into the reconstruction process has enabled great improvements in image quality, sharpness, and signal-to-noise ratio (SNR) in MRI and has consequently also accelerated acquisitions [15,16,17,18,19].

Malignancies of extremities are often located within direct proximity to small and vulnerable structures such as nerves, blood vessels, or tendons, which are essential for the function of the human locomotive system. Furthermore, high morphological resolution allows better lesion assessment as well as evaluation of tissue characteristics regarding benign and malignant criteria. Therefore, the purpose of this study is to investigate the technical applicability, image quality, and lesion detectability of deep learning-reconstructed MRI as compared to standard MRI in patients with tumors of the extremities.

## 2. Materials and Methods

### 2.1. Study Design

This monocentric, prospective, single institutional study was approved by the local institutional review board. Written informed consent was obtained from all study participants. The study was conducted in accordance with the ethical standards of the Declaration of Helsinki from 1964 and its latest revision in 2013. N = 23 patients who received an MRI examination of the extremities with a 1.5 T or 3 T scanner in our radiology department were included in the study.

### 2.2. MRI Examination Protocols

All MRI examinations were performed in clinical routine using 1.5 and 3 T scanners (MAGNETOM Vida, Prisma^fit^, Aera, and Avanto, Siemens Healthcare, Erlangen, Germany). Patients were examined in a supine position using a 32-channel spine coil and an 18-channel body coil array. The study protocol consisted of the following sequences: 1. Standard coronal T2w T2 Turbo inversion recovery magnitude (TIRM_Std_) with fat suppression. 2. Deep learning-accelerated T2w TSE (TSE_DL_) with spectral fat suppression based on a prototype. Detailed imaging parameters are displayed in Table 1 and Table 2. All MRI examinations were performed using a body-weight-adapted intravenous contrast agent injection (0.1 mmol/kg gadobutrol) (Gadovist, Bayer Healthcare, Berlin, Germany) with a flow rate of 1.5 mL/s followed by a saline flush of 20 mL.

### 2.3. Image Analysis

In a blinded random order reading, image analysis was performed independently by two radiologists, with 5 and 3 years of experience in MR imaging. The sequences were blinded for evaluation so that the readers did not know whether they were evaluating the T2w TIRM_Std_ or the T2w TSE_DL_. Due to the random order, a direct comparison, which might simplify the recognition of patterns in the sequence, should be avoided. For evaluation, a dedicated workstation (Centricity PACS RA1000; GE Healthcare, Milwaukee, WI, USA) was used. T2w TIRM_Std_ and T2w TSE_DL_ coronal were available for evaluation. Rating was performed using a Likert scale from 1 to 5, wherein 5 was the best and reading scores ≥3 were considered as sufficient for clinical use.

All images were rated for overall image quality (1, nondiagnostic; 2, highly reduced image quality; 3, moderate image quality 4, good image quality; 5, excellent image quality), noise levels (1, nondiagnostic; 2, high noise; 3, moderate noise; 4, little noise; 5, almost no noise), sharpness (1, nondiagnostic; 2, highly reduced sharpness; 3, moderate sharpness; 4, high sharpness; 5, excellent sharpness), contrast (1, nondiagnostic; 2, almost no contrast; 3, moderate contrast; 4, high contrast; 5, excellent contrast) and artifacts (1, nondiagnostic; 2, high level of artifacts; 3, moderate level of artifacts; 4, low level of artifacts; 5, almost no artifacts). Due to different fat saturation techniques, artifacts regarding fat saturation were not considered.

### 2.4. Lesion Assessment

A lesion was defined as a pathological finding of the extremities within the image, including the bones, soft tissues, and lymph nodes. Images were rated independently by the same two radiologists. The documentation of the lesion included the localization and the maximum diameter in millimeters. In addition, each lesion was evaluated regarding diagnostic confidence (1, nondiagnostic; 2, highly reduced diagnostic confidence; 3, moderate diagnostic confidence; 4, high diagnostic confidence; 5, excellent diagnostic confidence) and lesion detectability (1, nondiagnostic; 2, lesion barely detectable; 3, lesion moderately detectable; 4, lesion easily detectable; 5, lesion perfectly detectable), using a Likert scale from 1 to 5, wherein 5 was the best reading and scores ≥ 3 were considered as sufficient for clinical use.

### 2.5. Statistical Evaluation

Statistical analysis was performed using MedCalc Statistical Software version 18.10 (MedCalc Software bvba, Ostend, Belgium; http://www.medcalc.org (accessed on 1 July 2022); 2018) and jmp (MP^®^, Version 15 SAS Institute Inc., Cary, NC, USA, 1989–2019.). Data were tested for normal distribution using the Kolmogorov–Smirnov test. Parametric and non-parametric variables were recorded using median and interquartile range (IQR). We used the Wilcoxon signed-rank test for paired data of ordinal structure and non-normally distributed parametric variables. Numeric continuous, non-normally distributed data were tested using the Mann–Whitney U test. Inter- and intra-reader agreement was assessed by using Cohen’s kappa (0–0.20 = poor agreement, 0.21–0.40 = fair agreement, 0.41–0.60 = moderate agreement, 0.61–0.80 = substantial agreement, 0.81–1 = almost perfect agreement). *p*-values less than 0.05 were considered to indicate a significant difference. A Bland–Altman plot was used to illustrate the differences between the sequences in both readers. In a subgroup analysis, we compared the results of the patients who were examined with 3 T scanners and those who were examined with 1.5 T scanners.

### 2.6. Deep Learning Reconstruction

The deep learning reconstruction comprised an unrolled variational network, as used and detailed in Ref. [17]. The network architecture resembles an iterative parallel imaging reconstruction that is interleaved with regularization steps for intermediate images. As a key ingredient, these regularization steps are realized by CNNs whose parameterization was previously determined offline in a supervised training process using more than 10,000 representative images obtained from volunteers. The obtained parameterization was converted for use in a prototypical, scanner-integrated inference framework that was installed on the employed scanners. Inference time for a single slice in the actual deployment was about 3 s for CPU on average and 0.5 s for GPU.

## 3. Results

### 3.1. Patient Cohort

Twenty-three patients with a mean age of 60 ± 16 years and a range from 30 to 86 years were prospectively included in the study. Five patients underwent the MRI examination because of unclear findings in conventional X-ray-examinations (n = 3) or because of a newly diagnosed tumor of the extremities (n = 2) based on an X-ray or ultrasound examination. Eighteen patients received the examination as a follow-up of a known tumor of the extremities or after therapy of a local malignancy. A detailed listing of patients’ characteristics and diagnoses can be seen in Table 3.

### 3.2. Image Analysis

The test for interrater reliability showed an almost perfect agreement (κ = 0.886), so we decided to discuss only the results of the first reader. The detailed results of both readers are listed in Table 4 and Table 5.

### 3.3. Lesion Assessment

**Figure 1:**
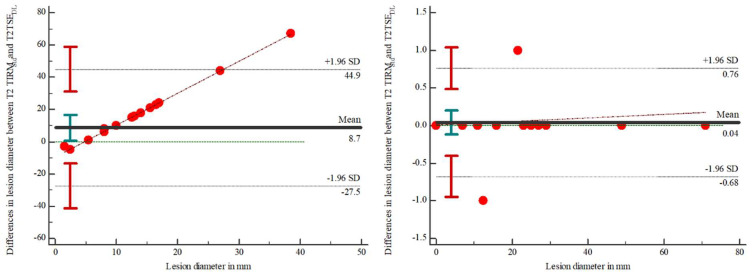
Bland-Altman plot to display the differences in the measurements of the lesion diameter between the T2-weighted TIRM_Std_ and the TSE_DL_ in reader 1 (**left**) and reader 2 (**right**).

In 12 of 23 MRI scans, a lesion could be detected. The analysis showed no differences between the number of detected lesions in both readers. The evaluation showed no statistically significant differences between T2 TIRM_Std_ (22 (13–29)) and T2 TSE_DL_ (22 (13–29)) for reader 1 (*p* = 0.982) and between T2 TIRM_Std_ (22 (13–29)) and T2 TSE_DL_ (22 (13–29)) for reader 2 (*p* = 0.895). Discrepancies regarding the lesion diameter between T2 TIRM_Std_ and T2 TSE_DL_ are illustrated in Figure 1. The results are illustrated in Table 5. Using Cohen’s kappa, interrater reliability was 0.945 for the T2 TIRM_Std_ and 1.0 for the T2 TSE_DL_.

### 3.4. Qualitative Image Analysis

**Figure 2:**
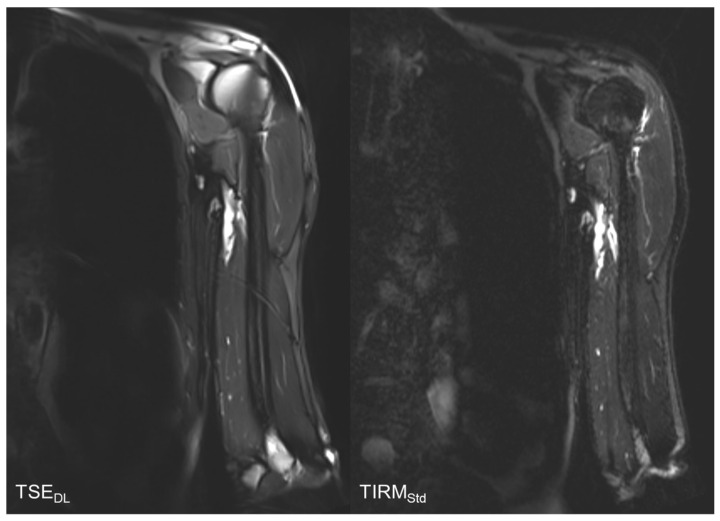
Comparison of T2-weighted TSE_DL_ and T2 TIRM_Std_. The T2 TIRM_Std_ shows a better image quality, contrast, and sharpness.

**Figure 3:**
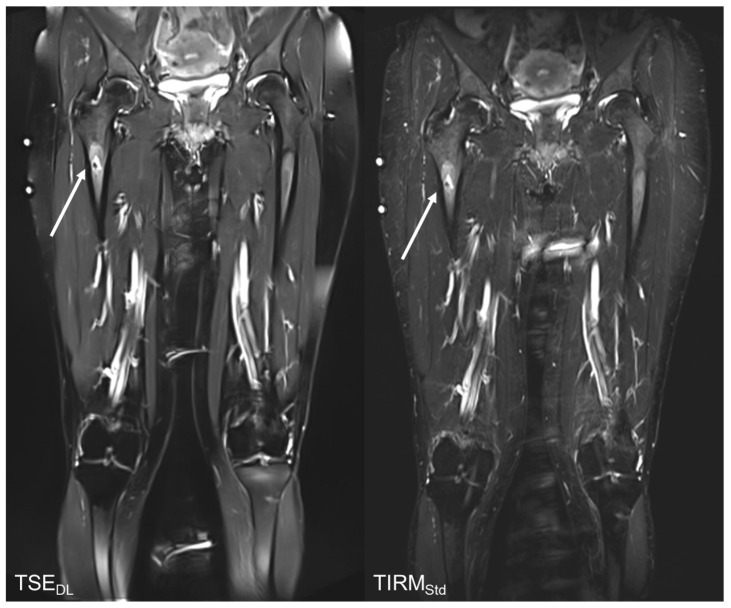
Follow up MRI examination of a 49-year-old with a T2w hyperintense lesion (arrows) of the right proximal femur, the finding would be compatible with enchondroma. The TSE_DL_ shows a better image quality, contrast, sharpness, and noise and thus, allows a better diagnostic confidence.

**Figure 4:**
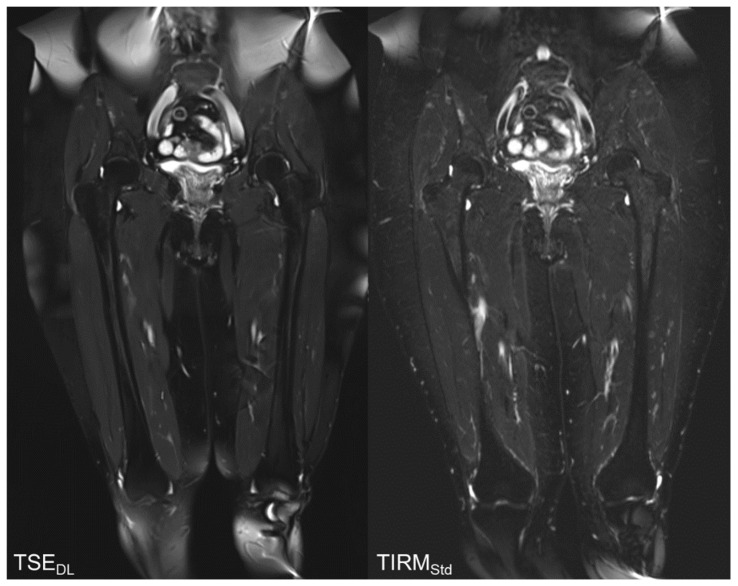
Follow-up MRI examination of an 81-year-old patient with Merkel cell carcinoma. As an incidental finding, the coronal TIRM_Std_ sequence shows a lipoma of the medial vastus muscle. The T2w TSE_DL_ shows a better image quality, noise, and sharpness than the TIRM_Std_.

**Figure 5:**
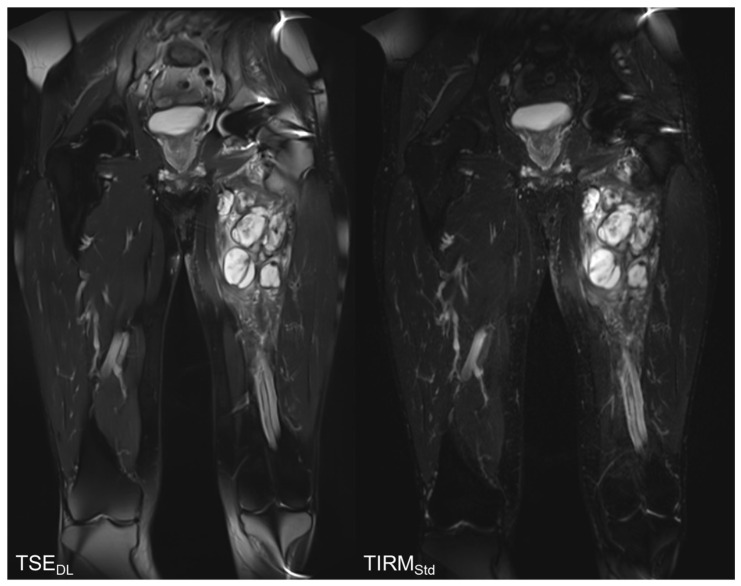
Follow-up MR examination of a 56-year-old male patient with histopathologically proven myxofibrosarcoma. T2w TSE_DL_ allows a better delineation of the conglomerate tumor of the left thigh due to better image quality, noise, sharpness, and contrast.

The results of the qualitative image analysis showed a significant improvement in the overall image quality, noise, sharpness, lesion detectability, and diagnostic confidence (each *p* < 0.001) for the T2 TSE_DL_ images, as compared to T2 TIRM_Std_. Concerning the level of artifacts, T2 TSE_DL_ was rated as slightly superior (*p* = 0.013). Imaging examples are displayed in Figure 2, Figure 3, Figure 4 and Figure 5.

### 3.5. Subgroup Analysis

In 6 patients, the examination was performed using a 3 T scanner while 17 patients were examined using a 1.5 T scanner. The subgroup analysis showed no significant differences for the qualitative results concerning the parameters image quality (*p* = 0.148–0.602), noise (*p* = 0.087–0.544), contrast (*p* = 0.2023–1.00), sharpness (*p* = 0.250–0.699), artifacts (*p* = 0.223–0.497), and diagnostic confidence (*p* = 0.243–0.424). Therefore, the field strength had no relevant impact on the reading results. 

### 3.6. Acquisition Time

#### 3.6.1. 1.5 Tesla Scanners

For the 1.5 T scanners, the average TA reduction for MRI of the upper extremities was 57% for the T2 TSE_DL_. For the lower extremities, time reduction was 59% for the T2 TSE_DL_.

#### 3.6.2. 3 Tesla Scanners

For the 3 T scanners, TA reduction for MRI of the upper extremities was 52% for the T2 TSE_DL_ and 59% for the T2 TSE_DL_ for MRI of the lower extremities.

## 4. Discussion

In this study, we investigated the technical feasibility of deep learning-accelerated sequences in MRI examinations of the extremities. We were able to show that the implementation of a deep learning-accelerated sequence leads to shorter acquisition times and better image quality, as compared to the conventionally used MRI sequence. Therefore, deep learning-accelerated T2-weighted fat-saturated imaging proved to be technically feasible while significantly improving TA, noise, contrast, sharpness, lesion detectability, diagnostic confidence, and image quality.

In some soft tissue tumors, imaging and clinical features allow a diagnosis without a biopsy, including myxoid liposarcoma, Baker’s cysts, neurofibroma, localized nodular synovitis, and cavernous hemangiomas. These lesions are characterized as determinate lesions that can be diagnosed with high specificity due to specific imaging features [20]. Due to its excellent soft tissue contrast, MRI is the best available option to characterize soft tissue lesions. Other imaging modalities, such as ultrasound with elastography, can help support the diagnosis [21]. Unfortunately, many malignant soft tissue lesions, especially those with low prevalence, are, due to their morphological resemblance, still frequently misdiagnosed as allegedly benign [21]. This often leads to a delay in therapy, which in turn may affect the final outcome for the patient. An improvement in image quality with a higher image resolution could lead to a higher specificity, and therefore, an earlier diagnosis.

In bone malignancies, early detection and treatment can significantly improve the prognosis [22]. In the most common primary bone malignancies, osteosarcoma, and Ewing sarcoma, differentiation might be challenging due to a similar signal behavior and appearance [23]. Furthermore, there is a huge variety of benignant bone lesions that might be difficult to distinguish from malignancies [24]. Hence, a good image quality to precisely evaluate the tumor structure is essential.

Regarding the results of our study, the T2 TSE_DL_ showed an excellent image quality, superior to conventionally used MRI sequences, while improving TA. In a rather small study population, no differences were found in the number of detected pathologies. Lesion detectability and diagnostic confidence proved to be better in the novel deep learning-accelerated sequence; thus, a high detection rate may be assumed, with concomitantly improved image quality, possibly even better than in currently used sequences. Nevertheless, further investigation will be necessary to determine the diagnostic accuracy of deep learning-accelerated sequences in daily clinical practice.

One of the most important advantages of deep learning-based algorithms in image reconstruction is the possible shortening of the TA [25,26,27]. One concern associated with a shortened acquisition time is that it might lead to an increased occurrence of artifacts. However, extremities are less susceptible to motion artifacts than other anatomical structures such as the abdominal and thoracic organs, as imaging is not dependent on breath-hold acquisitions. Our study could prove that T2 TSE_DL_ does not lead to a more frequent emergence of artifacts. The improvement in TA could also imply a better tolerance of MRI examinations in children and young adolescents, who are more likely to develop soft tissue malignancies of the extremities [28]. Better image quality and higher SNR could help to improve the sensitivity and specificity of MRI in extremity tumors. Additionally, a shortened TA would improve the total time required for MRI examinations and, thereby, increase the availability of MRI examinations in the healthcare sector and improve the economic efficiency of MR imaging.

The novelty of our study is that the technical feasibility and the clinical applicability of deep learning-accelerated imaging were tested for the first time in extremity tumors. Although deep learning MRI still has an exploratory aspect, the results indicate numerous advantages that will allegedly make integration into clinical practice inevitable.

There are some limitations to be considered. Firstly, only one sequence, namely the T2w TSE_DL,_ was compared to the conventionally used T2w TIRM_Std_. No further characterization of lesions was assessed; thus, the final impact on the specificity remains unclear. In addition, only a small cohort of 23 patients was included. In conclusion, our study shows the technical feasibility of deep learning-based T2w TSE_DL_, which proved to be superior to conventional T2w TIRM with regard to all examined image parameters. Additionally, novel deep learning-based sequences allow a significant time reduction of more than a factor of two.

## Figures and Tables

**Table 1:** Acquisition parameters for lower extremities at 1.5 T and 3 T.

Sequence	T2 TIRM_Std_ Coronal	T2 TSE_DL_ Coronal	Sequence	T2 TIRM_Std_ Coronal	T2 TSE_DL_ Coronal
TE [ms]	71	71	TE [ms]	74	74
TR [ms]	5440	5880	TR [ms]	6030	6200
FA [°]	150	140	FA [°]	150	140
TA [min:s]	2:34 min	1:06 min	TA [min:s]	2:50 min	1:10 min
Slice thickness [mm]	5.0	5.0	Slice thickness [mm]	5.0	5.0
FOV (mm^2^)	460 x 460	460 x 460	FOV (mm^2^)	460 x 460	460 x 460

T2w: T2-weighted; TE: time of echo; TR: time of repetition; FA: field angle; TA: time of acquisition; FOV: field of view.

**Table 2:** Acquisition parameters for upper extremities at 1.5 T and 3 T.

Sequence	T2 TIRM_Std_ Coronal	T2 TSE_DL_ Coronal	Sequence	T2 TIRM_Std_ Coronal	T2 TSE_DL_ Coronal
TE [ms]	71	71	TE [ms]	74	74
TR [ms]	5440	6060	TR [ms]	6030	6200
FA [°]	150	140	FA [°]	150	140
TA [min:s]	2:34 min	1:14 min	TA [min:s]	2:50 min	1:10 min
Slice thickness [mm]	5.0	5.0	Slice thickness [mm]	5.0	5.0
FOV (mm^2^)	500 x 500	500 x 500	FOV (mm^2^)	500 x 500	500

T2w: T2-weighted; TE: time of echo; TR: time of repetition; FA: field angle; TA: time of acquisition; FOV: field of view.

**Table 3:** Patient cohort.

Patients (Male/Female), n	23 (16/7)
Age, mean ± SD (range), y	total: 60 ± 16 (30–86)
	male: 55 ± 15 (30–81)
	female: 70 ± 12 (50–86)
Diagnosis, n	Liposarcoma, 5
	Neurinoma, 2
	Leiomyosarcoma, 2
	Lipoma, 2
	Enchondroma, 2
	Unclear mass, 2
	Unclear symptoms needing further specification, 2
	Myxofibrosarcoma, 2
	Pleomorphic sarcoma, 1
	Not otherwise specified sarcoma, 1
	Spindle cell sarcoma, 1
	Ewing sarcoma, 1

**Table 4:** Detailed results of the image analysis.

	Reader 1			Reader 2		
	T2 TIRM_Std_ Median (IQR)	T2 TSE_DL_ Median (IQR)	*p*-Value	T2 TIRM_Std_ Median (IQR)	T2 TSE_DL_ Median (IQR)	*p*-Value
Overall Image Quality						
IQ	4 (3–4)	5 (5–5)	<0.001	4 (4–4)	5 (5–5)	<0.001
Noise	4 (3–4)	5 (5–5)	<0.001	4 (3–4)	5 (4–5)	<0.001
Contrast	4 (3–4)	5 (5–5)	<0.001	4 (4–4)	5 (4–5)	<0.001
Sharpness	4 (3–4)	5 (5–5)	<0.001	4 (3–4)	5 (5–5)	<0.001
Artifacts	4 (4–4)	5 (4–5)	0.013	4 (4–4)	4 (4–5)	0.542

IQ = image quality; DC = diagnostic confidence; IQR = interquartile range. Detailed results of the image analysis of both readers for T2w sequences.

**Table 5:** Lesion assessment.

	Reader 1			Reader 2		
	T2 TIRM_Std_ Median (IQR)	T2 TSE_DL_ Median (IQR)	*p*-Value	T2 TIRM_Std_ Median (IQR)	T2 TSE_DL_ Median (IQR)	*p*-Value
Lesion Assessment						
Lesion size	22 (13–29)	22 (12–29)	0.982	22 (13–29)	22 (12–29)	0.797
Lesion detectability	4 (4–5)	5 (5–5)	<0.001	4 (4–5)	5 (5–5)	0.003
Diagnostic confidence	4 (4–4)	5 (5–5)	<0.001	4 (4–4)	5 (5–5)	0.003

IQR, interquartile range. Lesion assessment of the T2-weighted sequences.

## Data Availability

The data presented in this study are available on request from the corresponding author. The data are not publicly available due to privacy and ethical reasons.

## Abbreviations

CT	Computed tomography
CNN	Convolutional neural network
DL	Deep Learning
FA	Flip Angle
pc	post-contrast
SNR	Signal-to-Noise Ratio
Std	Standard
T2 TSEDL	T2-weighted turbo deep learning accelerated spin echo sequence
TA	Time of Acquisition
T2w	T2-weighted
tra	transversal
TSE	Turbo spin echo
TSEStd	standard Turbo spin echo sequence
TIRM	Turbo inversion recovery magnitude
TIRMStd	standard Turbo inversion recovery magnitude sequence
